# Multimodality Imaging in the Diagnostic Work-Up of Endocarditis and Cardiac Implantable Electronic Device (CIED) Infection

**DOI:** 10.3390/jcm9072237

**Published:** 2020-07-14

**Authors:** Nicola Galea, Francesco Bandera, Chiara Lauri, Camillo Autore, Andrea Laghi, Paola Anna Erba

**Affiliations:** 1 Department of Experimental Medicine, “Sapienza” University of Rome, 00161 Rome, Italy; 2 Heart Failure Unit, Cardiology University Department, IRCCS Policlinico San Donato, Piazza Malan, 1, San Donato Milanese, 20097 Milan, Italy; francesco.bandera@unimi.it; 3 Department of Biomedical Sciences for Health, University of Milano, Via Luigi Mangiagalli, 31, 20133 Milan, Italy; 4 Nuclear Medicine Unit, Department of Medical-Surgical Sciences and of Translational Medicine, “Sapienza” University of Rome, 00161 Rome, Italy; chialau84@hotmail.it; 5 Department of Clinical and Molecular Sciences, “Sapienza” University of Rome, 00189 Rome, Italy; camillo.autore@uniroma1.it; 6 Radiology Unit, Department of Medical-Surgical Sciences and of Translational Medicine, “Sapienza” University of Rome, 00189 Rome, Italy; andrea.laghi@uniroma1.it; 7 Department of Nuclear Medicine, Department of Translational Research and New Technology in Medicine, University of Pisa, 56126 Pisa, Italy; p.erba@med.unipi.it; 8 Medical Imaging Center, Department of Nuclear Medicine and Molecular Imaging, University of Groningen, University Medical Center Groningen, 9713 Groningen, The Netherlands

**Keywords:** Infective endocarditis, echocardiography, multimodality imaging, computed tomography, magnetic resonance imaging, nuclear imaging, positron emission tomography, endocarditis team

## Abstract

Infective endocarditis (IE) is a serious cardiac condition, which includes a wide range of clinical presentations, with varying degrees of severity. The diagnosis is multifactorial and a proper characterization of disease requires the identification of the primary site of infection (usually the cardiac valve) and the search of secondary systemic complications. Early depiction of local complications or distant embolization has a great impact on patient management and prognosis, as it may induce to aggressive antibiotic treatment or, in more advanced cases, cardiac surgery. In this setting, the multimodality imaging has assumed a pivotal role in the clinical decision making and it requires the physician to be aware of the advantages and disadvantages of each imaging technique. Echocardiography is the first imaging test, but it has several limitations. Therefore, the integration with other imaging modalities (computed tomography, magnetic resonance imaging, nuclear imaging) becomes often necessary. Different strategies should be applied depending on whether the infection is suspected or already ascertained, whether located in native or prosthetic valves, in the left or right chambers, or if it involves an implanted cardiac device. In addition, detection of extracardiac IE-related lesions is crucial for a correct management and treatment. The aim of this review is to illustrate strengths and weaknesses of the various methods in the most common clinical scenarios.

## 1. Introduction

Infective endocarditis (IE) is a complex pathological entity with various clinical presentations, whose diagnosis may be challenging as based on a combination of multiple clinical, biological, and imaging criteria [1,2]. Similar difficulties are encountered when the infection is suspected in patients with prosthetic valve (PV) or cardiac implantable electronic device (CIED).

The key elements for disease characterization are to identify the pathogen in the blood, to detect vegetation on the cardiac valves (native or prosthetic) or adhering to CIED, and to assess local complication or distant embolization.

In this perspective, the choice of the most appropriate diagnostic imaging tool can play a crucial role in both confirming the diagnosis and guiding the treatment.

Results of imaging need to be multidisciplinarily discussed within the Endocarditis Team to optimize its value, thus guiding proper therapeutic strategies, eventually improving patient care.

The aim of the present review was to provide an overview of the pros and cons of the different imaging techniques to answer specific questions in the most common clinical scenarios.

## 2. Clinical Diagnosis: From the Duke Criteria to the European Society of Cardiology (ESC) 2015 Criteria and the Novel 2019 International CIED Infection Criteria

Imaging plays a key role in both the diagnosis and management of IE. Imaging-derived parameters are also useful for the prognostic assessment of patients with IE, for its follow-up under therapy, and during and after surgery. Imaging findings are part of the diagnostic criteria used in clinical practice to reach a diagnosis. The diagnostic strategy proposed by Durack et al. [3] (the Duke criteria) combined echocardiographic findings with clinical and microbiological data. Three echocardiographic findings were considered to be major criteria for the diagnosis of IE: (a) Presence of vegetations, (b) presence of abscesses, or (c) presence of a new dehiscence of a valvular prosthesis. Other abnormal echocardiographic findings not fulfilling these definitions were considered minor criteria. This classification has an overall sensitivity of approximately 80% when the criteria are evaluated at the end of patient follow-up in epidemiological studies. However, the Duke criteria show a lower accuracy for early diagnosis in clinical practice, especially in the case of prosthetic valve endocarditis (PVE) and CIED-related infective endocarditis (CIED-IE), for which echocardiography is normal or inconclusive in up to 30% of cases [4].

Therefore, more recent guidelines [2] incorporate the use of multimodality imaging, including molecular imaging techniques, to integrate the traditional diagnostic criteria in order to fill in such uncertainty gap with information on the biochemical burden of these infections.

Abnormal activity around the prosthetic valve detected by fluoro-18-fluorodeoxyglucose ((^18^F)FDG) positron emission tomography/computed tomography (PET/CT) or radiolabeled white blood cells (WBC) scintigraphy with single-photon emission computed tomography/computed tomography (SPECT/CT) is considered a major criterion for the diagnosis of IE according to ESC guidelines published in 2015. Both techniques are currently applied in the diagnostic workup of IE and CIED with two main indications: Confirming the presence of infection and identification of septic emboli. By this approach, a substantial reduction in the rate of misdiagnosed IE has been demonstrated. In general, (^18^F)FDG PET/CT is characterized by higher spatial resolution and sensitivity, better image quality, and shorted acquisition times compared to WBC scan. In contrast, WBC scintigraphy is more specific than (^18^F)FDG PET/CT, being able to achieve a differential diagnosis between a sterile inflammation, as observed early after surgery. Therefore, WBC imaging should be preferred in all the situations that require higher specificity or in case of inconclusive (^18^F)FDG PET/CT. Major drawbacks of WBC include the relatively complex and time-consuming labeling procedure that requires a particular equipment, the handling of potentially infected blood, and longer acquisition times. At the moment, no sufficient literature exists in support of one of these imaging modalities rather than another. Therefore, the choice mainly depends on local center availability and expertise, including the presence of SPECT/CT equipment, which is the gold standard for this application, waiting list, and isolated strains (*Candida* spp. and *Enterococcus* spp. may provide false negatives scan due to their ability to create biofilm that impairs granulocytes accumulation in the infected site). In addition, the choice between WBC scintigraphy and (^18^F)FDG PET/CT remains a prerogative of the Endocarditis Team’s discussion. Important parameters to be considered for a proper positron emission tomography (PET) reading are the location, pattern, and intensity of the (^18^F)FDG. The uptake can be classified as intravalvular (in the leaflets), valvular, or perivalvular [5], even though it should be noted that the intravalvular location is rare. Focal and heterogeneous uptake is consistent with an infected valve. A typical location for abscesses in PVE is the aorto-mitral intervalvular fibrosa, but abscesses can develop in any region in contact with prosthetic material. The probability of infection increases with the intensity of the (^18^F)FDG uptake; however, several factors may influence (^18^F)FDG avidity and, therefore, they must be carefully considered for the correct interpretation of a PET/CT scan. For example, prolonged antibiotic therapy, and consequently reduced inflammatory burden, or small vegetations may result in a false negative PET/CT scan. Conversely, recent implantation, especially of mechanical valves, some types of surgical adhesives, or inadequate myocardial suppression usually shows enhanced (^18^F)FDG uptake. Moreover, active thrombi, vasculitis, primary cardiac tumors, or cardiac metastasis could mimic a focal uptake, thus representing additional confounding factors [5]. In these cases, a WBC scintigraphy could be nullifying. If PET/CT acquisition is combined with a cardiac computed tomography angiography (CCTA), the metabolic findings provided by the (^18^F)FDG uptake distribution and intensity might be added to the anatomic findings already described for CCTA within a single imaging procedure [6]. The advantages of combining (^18^F) FDG PET/CT with CCTA include the identification of a larger number of anatomic lesions and clarification of the indeterminate studies by echocardiography [6,7]; furthermore, it assumes a great value in specific clinical situations such as in patients with aortic grafts or with congenital heart disease who have complex anatomy, as their surgical treatment often requires implantation of a large amount of prosthetic material.

In case of CIED-IE, which includes pacemakers and implantable cardioverter defibrillators, the presence of (^18^F)FDG uptake located on or alongside a lead and that persists on non-attenuation-corrected (NAC) images, is considered consistent for an infectious process according to the very recently published Novel 2019 International CIED Infection Criteria [8].

## 3. Multidisciplinary Approach of Endocarditis Team

Given the complexity of both diagnosis and therapeutic approach of IE and CIED-IE, no practitioner would be able to manage alone such diseases, being that they are characterized by a wide panel of signs and symptoms and clinical presentations. Therefore, the collaboration between different specialists that look at the same problem from different points of view is crucial for the successful treatment of such infections. A multidisciplinary approach had already shown several advantages in other clinical contexts, for example, in valve diseases, as also recommended by American Heart Association/American College of Cardiology [9]. Following this view, ESC guidelines on IE published in 2015 [2] underlined the need to refer such kind of patients to specialized centers with immediate access to diagnostic procedures and surgical facilities. A pivotal aspect of this approach is represented by the “Endocarditis Team” that involves cardiologists, cardiac surgeons, imaging specialists, microbiologists, infective diseases specialists, neurosurgeons, and other specialists involved in a case-by-case scenario, each one with his or her specific expertise and competence, aiming to ensure best management for the patients, especially in complicated scenarios. Communication among these different specialists plays an important role and, therefore, cases should be regularly discussed during meetings in order to achieve a consensus on the most appropriate treatment for each patient and to define the type and duration of follow-up.

Beside “multidisciplinarity”, “multimodality” and “multitracers” are the other two key words that are becoming increasingly important for the management of IE and CIED-IE, thus configuring the so-called “3M” approach to cardiovascular infections [10]. It underlines the importance to appeal to several imaging modalities and strategies that are able to study different aspects of the same problem and to provide relevant information for the clinicians.

## 4. Left Heart Native Valve IE

### 4.1. Main Clinical Characteristics

IE of the left heart valves is an infective process affecting the endothelial surface of the aortic or mitral valve. In the general population, the incidence is 3–10 per 100,000 patients per year, but it can reach 20–60 per 1000 patients/year in the case of recurrence [11]. In the last decades, the peak of age has been shifted toward the elderly [12]. The great clinical impact of IE relies on the high in-hospital and six months’ mortality rate of 20% and 25–30%, respectively [13,14].

The main valve-related risk factor is the presence of degenerative (fibro-calcific disease in high-income countries or rheumatic disease in low-income countries) or congenital (mitral prolapse, bicuspid aortic valve) abnormalities, determining abnormal flow and increased shear stress on the endothelial surface. The host-related risk factors are the clinical conditions determining systemic immunodepression, such as diabetes and cancer [2].

Clinical presentation is typically characterized by the signs and symptoms incorporated in Duke criteria (fever, vascular and immunological phenomena). Nevertheless, elderly patients can have atypical presentations characterized by the absence of fever, pre-existing heart murmurs, or blunted rise of inflammatory markers, resulting in more difficult diagnostic work-up.

The microbiological isolation, by means of repeated blood cultures, and the demonstration of vegetation at echocardiography are the cornerstones of diagnosis, according to Duke criteria [1]. The overall criteria provide a definite, possible, or rejected diagnosis, according with the clinical probability defined by the combination of them. Left heart valves are generally well explored by ultrasound; however, conditions such as age-related, fibro-calcific degeneration, low-quality acoustic window, or pre-existing valve disease can challenge the identification of vegetation. Nevertheless, in the case of possible IE (according with Duke criteria) or high suspicious IE, further diagnostic work-up is indicated using CCTA scan [2].

The main complications of left-sided IE are heart failure (HF), systemic embolism, and uncontrolled infection [15]. HF is the most frequent complication and can be observed in up to 60% of patients with aortic valve IE, being a predictor of in-hospital, six and 12 months’ mortality [16]. HF represents an indication for early surgery also in case of hemodynamic instability. The perivalvular extension and the presence of difficult-to-treat organisms are the main causes of uncontrolled infection, representing an additional indication for early surgery [17]. Finally, systemic embolism is a very common and disregarded complication (up to 50% of cases) requiring specific diagnostic work-up when it is suspected, especially in patients with persistent or recurrent fever and bacteremia or symptomatic patients with recent neurological events. The vegetation size and mobility, age, diabetes, infection by *Staphilococcus aureus*, atrial fibrillation, and previous embolism are the main risk factors for embolism occurrence. During the first two weeks of antibiotic treatment, the risk of embolism is higher, requiring a strict clinical monitoring [18].

### 4.2. When to Ask for Transthoracic Echocardiography (TTE) and When to Ask for Transoesophageal Echocardiography (TOE)

**Figure 1:**
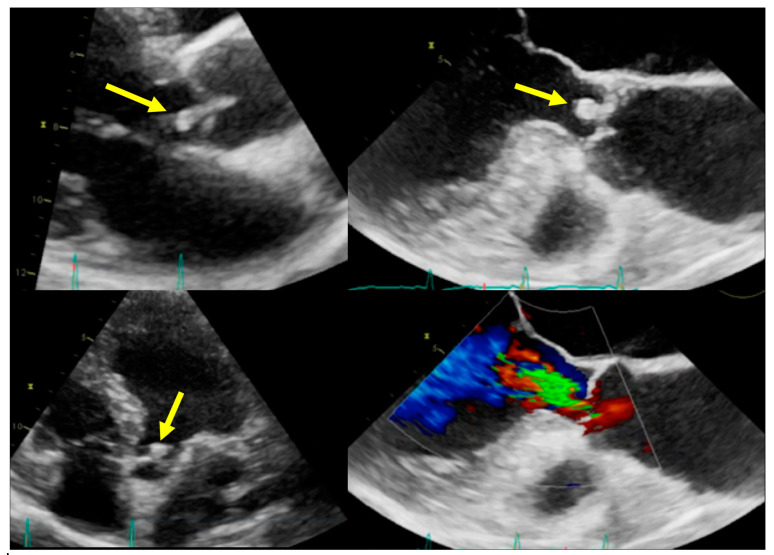
Infective endocarditis (IE) on aortic valve. Top and bottom left: Parasternal long axis and apical four-chamber transthoracic echocardiography (TTE) showing vegetation (arrows) in the left ventricle outflow tract. Top and bottom right: Transoesophageal echocardiography (TOE) showing the vegetation and the related valvular regurgitation.

All patients with a diagnostic suspicion of left heart IE should receive transthoracic echocardiography (TTE). TTE is the first-line diagnostic step and is aimed at the direct identification of the vegetation and of the related damages of the valves (Class of recommendations I, Level of evidence B) [2]. The presence of abscess or pseudoaneurysm and new dehiscence of prosthesis are additional major Duke criteria. In both aortic and mitral IE, acute regurgitation can develop, especially when the causative germs are *Staphilococci* (Figure 1).

Serial monitoring is required, even when the diagnosis has been achieved [19].

TTE has a limited sensitivity (ranging from 50 to 60%) mainly related to the anatomical or technical limitations; therefore, transoesophageal echocardiography (TOE) is strongly indicated in case of nondiagnostic or negative TTE (Class of recommendations I, Level of evidence B) [2]. TOE should also be considered in case of positive TTE to obtain a more accurate characterization of the vegetation, to exclude the complications, and to evaluate the vegetation sizes. Globally, the diagnostic sensitivity of TOE is about 85–90% [20]. According to the Euro-Endo registry, TTE has been performed in 91% of cases, while TOE in 53% of native valves’ IE [21]. However, some heterogeneity in the diagnostic workup has been reported, with some countries having a more extensive use of imaging, possibly associated with a relatively low mortality [22]. Globally, the data confirm the current role of TTE as a first-line test, while TOE is still limited to selected cases.

**Figure 2:**
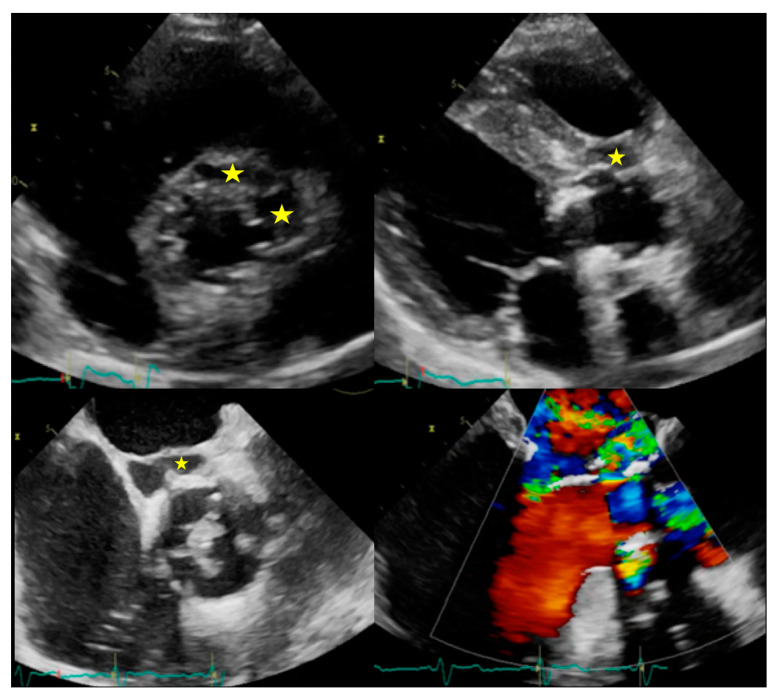
Complications of IE. Top: Example of peri-aortic abscess (stars) with large anechoic cavity surrounding the biological prosthesis (left: TTE with parasternal short axis view, right: TTE with parasternal long axis view). Bottom: Example of peri-aortic pseudoaneurysm (star) with large cavity communicating with cardiovascular lumen (left: TOE short axis view showing large vegetation on prosthesis cusps, right: TOE log axis view with color Doppler showing flow into the perivalvular cavity).

Both TTE and/or TOE are indicated during the follow up to identify clinically evident (Class of recommendations I, Level of evidence B) or silent (class Class of recommendations IIa, Level of evidence B) complications, as well as to re-evaluate the patient at the completion of antibiotic therapy (Class of recommendations I, Level of evidence C, Figure 2) [2].

### 4.3. Role of CCTA in Diagnosing IE and Local Complications

CCTA offers valve imaging with high spatial and temporal resolution and has been established as a valid imaging option when TTE is not definitive or is limited (e.g., poor acoustic window, unclear findings, extensive calcification) [2] or when it does not show any abnormality even though IE is clinically suspected.

**Figure 3:**
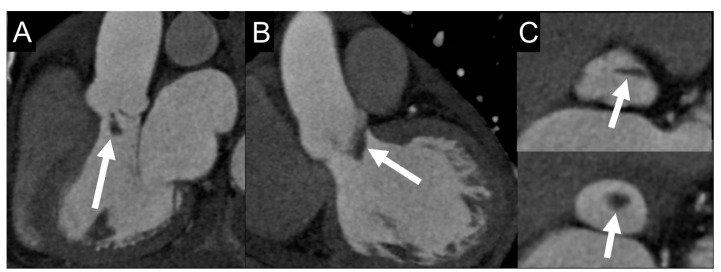
Vegetation on cardiac computed tomography angiography (CCTA) images. A 69-year-old man with fever and IE. Multiplanar CCTA reconstructions on three-chamber (**A**) and coronal (**B**) views show a 2-cm, hypodense, club-shaped, soft-tissue oscillating mass (arrows) attached to the ventricular side of aortic valve leaflets, which appear floating in the lumen of the left ventricular outflow tract in reconstructed axial valve planes (**C**). Sensitivity of CCTA in detecting vegetation ranges from 52.8% for small lesion to 94.4% for larger ones (>10 mm) [24].

On CCTA images, vegetations may appear as leaflet thickening or irregularly shaped soft-tissue oscillating masses, adherent to the valve or endomyocardial surface (Figure 3) [23].

CCTA can play a role in assessing the embolic risk as several factors, in particular vegetation size >10 mm and mobility, are predictors of embolic events [2].

CCTA is inferior to TOE in detecting small vegetations (<2 mm) and valve perforations, but is superior in the assessment of the perivalvular extent of the disease such as abscesses, pseudoaneurysms, and fistulas [25], with a sensitivity of 100% using surgery as a reference standard [26].

Abscesses are seen on CCTA images as a perivalvular, low-attenuated fluid collection, bordered by thickened inflammatory tissue, which typically enhance after contrast administration or irregular inhomogeneous tissue adjacent to the fluid. CCTA imaging may identify the abscess extension into surrounding structures, such as into the interatrial septum or mitral-aortic intervalvular fibrous body, which may have implications in surgical planning.

**Figure 4:**
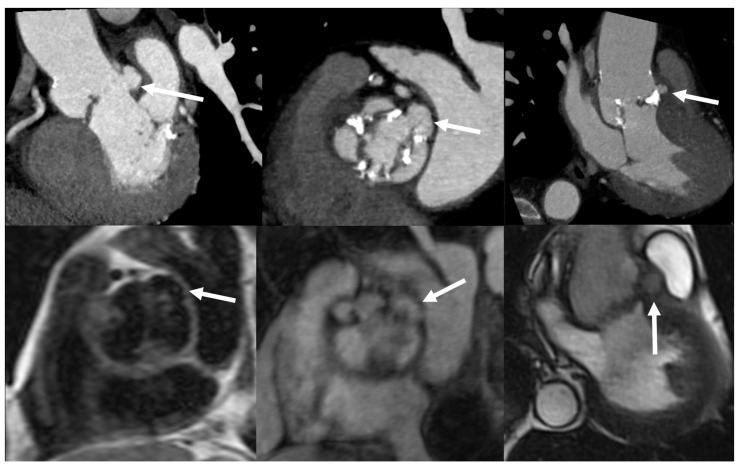
Paravalvular abscesses. CCTA (top images) multiplanar reconstructions and Cardiovascular Magnetic Resonance (CMR) images (bottom images: T1-weighted Turbo Spin Echo on the left, contrast-enhanced T1-weighted Gradient Echo 3D image in the middle and cine-CMR on the right) show a diffuse, partially calcified, thickening of the aortic valve leaflets in a 58-year-old man with *Staphilococcus aureus* IE and bicuspid valve. IE was complicated by the formation of small perivalvular abscesses, which, following the opening of their contents in the lumen, appear as little saccular outpouchings (arrows).

Pseudoaneurysms appear on CCTA as contrast-containing outpouchings of endocardial wall, freely communicating with cardiac chambers or the aortic root, usually located at the paravalvular or periannular regions, possibly extending to the myocardium or pericardium (Figure 4).

Perforation of the aortic or mitral valve leaflets is visible on CCTA as focal defect and its detection may be helped by the application of 3D volume-rendering reconstructions [27].

Furthermore, CCTA can non-invasively rule out coronary artery disease before surgery by avoiding invasive coronary angiography, which has an intrinsic procedural risk of systemic embolism of valve vegetations or aortic wall perforation, especially in patients with extensive involvement of the aortic valve by IE [25].

### 4.4. When to Ask for Nuclear Imaging 

The value of (^18^F)FDG PET/CT and WBC imaging is limited in native valve IE, in which the sensitivity is too poor to recommend its routine use [28,29,30]. However, in the case of native valve IE, (^18^F)FDG PET/CT is useful for the detection of distant embolic events, a condition currently considered a minor criterion in the 2015 ESC guidelines [2]. Indeed, whole body (^18^F)FDG PET/CT offers the possibility to evaluate, with a single imaging modality, both cardiac and extra-cardiac foci, thus allowing the identification of eventual “metastatic” sites of infection with high sensitivity (see below). In addition, (^18^F)FDG PET/CT imaging is also useful in the identification of the portal of entry (POE), fundamental to minimize the risk of recurrence.

### 4.5. How to Search for Embolisms

As embolic events complicate a large number of IE patients [2,18], especially during the first week of therapy, and may have a dramatic impact on patient prognosis, their prompt recognition is required. Septic emboli or vascular phenomena may be totally silent in 20–50% of cases [2,31], especially those affecting the splenic or cerebral circulation, which are the most frequent sites of embolism in left-sided IE.

The evidence of septic emboli or vascular phenomena on imaging is included as minor Duke criterion for IE diagnosis [1,2]. Thus, systematic, whole-body contrast-enhanced computed tomography (CT) and/or [^18^F]FDG PET/CT and cerebral CT or magnetic resonance imaging (MRI) should be considered in both suspected and definite IE.

Cerebral imaging is mandatory for any suspicion of neurological complication and brain MRI is more sensitive than CT for detection of cerebral lesions (mostly ischemic and, less frequently, abscessual or hemorrhagic). However, in unstable or uncooperative patients, CT may be preferable because it is faster and easily feasible. MRI or nongated, contrast-enhanced CT angiography should be included in the imaging protocol in order to rule out vascular lesions such as embolic occlusion or mycotic aneurysm [31].

Contrast-enhanced, whole-body CT and (^18^F)FDG PET/CT scan have high diagnostic accuracy for splenic abscesses, metastatic infection of other abdominal parenchymal organs, and vascular lesions (splanchnic or peripheral septic emboli). Nevertheless, the administration of iodinated contrast media should be avoided or limited in patients with renal impairment, especially when antibiotic with nephrotoxic effect is used.

### 4.6. Role of Cardiovascular Magnetic Resonance (CMR) in Diagnosing IE and Local Complications

Although CMR may detect vegetations, abscesses, or pseudoaneurysms in IE, its role in the initial diagnosis is limited and it is not included in ESC 2015 modified diagnostic criteria.

In particular, CMR could be preferred to CCTA in the presence of renal insufficiency as vegetations and local complications may be well depicted on noncontrast CMR images (Figure 4) or in pediatric patients, to avoid radiation exposure. Vegetations, in particular, appear as low signal nodules or floating filaments adherent to the leaflets surface or endocardium.

CMR with late, enhanced imaging may help to detect myocarditis, which is frequently associated with abscess formation or immune reaction [2].

### 4.7. Diagnostic Workflow Summary

• The initial assessment of suspected left-sided native IE is based on Duke criteria.
• The patient must receive TTE, TOE, and blood cultures.
• If IE is rejected and the suspicious is low, no more investigations are needed.
• If the diagnosis is definite, the patient should be investigated for silent embolism using CT or PET/CT scan, as well as MRI for cerebral involvement (embolism or hemorrhage), according with clinical status.
• In the case of possible IE or rejected IE but high suspicion there is indication for repeating new TTE/TEE and blood cultures, further investigations, such as whole-body, contrast-enhanced CT or PET/CT scan, should be considered to detect silent embolism or metastatic infections and, therefore, to reclassify the patient, according with ESC 2015 modified diagnostic criteria [2].

## 5. Right Heart Native Valve IE

### 5.1. Main Clinical Characteristics

Right heart IE represents 5–10% of all IE and is typically associated with intravenous drug use, congenital heart disease, intravascular catheters, and immunodepression states (such as HIV infection) [32]. The tricuspid valve is predominantly involved, especially in active intravenous drug users, whose 5-year survival is 50% in case of surgical treatment [33].

The clinical presentation is frequently characterized by respiratory symptoms resulting from pulmonary emboli, pneumonia, and abscess formation. Anemia and microscopic hematuria can typically be present when the tricuspid valve is involved [34]. Systemic emboli and sepsis are potential complications accounting for a worse prognosis. The overall mortality has been reported to be as high as 10.2%, with a surgical mortality of 7.8%, both related to the risk factors, vegetation size, and location [35].

In intravenous drug users, *Staphylococcus aureus* is the most common germ for IE, localizing on the tricuspid valve and accounting for a 16% mortality rate [36]. IE related to the presence of central venous catheter is predominantly caused by *Staphylococcus aureus* (54.6%), coagulase-negative *Staphylococcus* (37.5%), *Candida* species (16.6%), and *Enterococcus* (12.5%) [37].

A specific subgroup of IE is represented by the infections affecting people with congenital heart disease (CHD). The incidence is 15–140 times higher than in the general population and the reported mortality ranges between 4 to 10% [38,39]. The diagnostic management is substantially similar to that of native valve IE but it is challenged by the morphological complexity of some CHD. A specific risk factor for both early and late IE is the presence of valve-containing prosthetics, a condition frequently encountered in complex CHD that predisposes a patient to a greater long-term risk [40].

### 5.2. When to Ask for TTE and When to Ask for TOE

As for left-side IE, TTE is the first-line diagnostic tool allowing for a good evaluation of the tricuspid valve, especially when using off-axis plans. The pulmonary and Eustachian valves are more challenging to explore with TTE and, therefore, they frequently require trans-esophageal approach. The vegetation size (highly correlated with the risk of recurrent embolism), the worsening of regurgitation, the degree of congestion, and the presence of HF represent a class IIaC indication for surgical treatment and require systematic echocardiographic assessment during follow up [2]. In case of suspected IE in CHD adult patients, TTE is often inadequate for a complete visualization of cardiac structures, especially in complex malformations and, therefore, TOE is required to provide a complete assessment [39]. In children, TTE generally allows for a correct assessment.

### 5.3. Role of CCTA in Diagnosing IE and Local Complications

Similarly to what is described for the left valves, CCTA play a role in detecting the vegetations and local complications, such as perivalvular extension of infection, transvalvular fistula, perivalvular abscess, and pseudoaneurysm [41]. In particular, the evaluation of the pulmonary and tricuspid valves by TTE is limited by the poor acoustic window; therefore, CCTA can offer an added value when echocardiography is not definitive [31].

The CCTA protocol should guarantee an adequate contrast enhancement of the right chambers, whereas the standard CCTA protocol for the assessment of the coronary tree and left chambers comprises the complete washing of the right cavities with iodine.

### 5.4. When to Ask for Nuclear Imaging

As for left side native valve IE, the use of nuclear imaging in native valve IE is limited and mainly addressed to the detection of pulmonary embolism and for the identification of POE (see below).

### 5.5. How to Search for Embolisms

Right-side IE and CIED-IE are associated to lung metastatic infection. Pulmonary infectious emboli should always be sought when the IE diagnosis is possible, according to modified Duke criteria, or rejected despite the high clinical suspicion, as it is considered a minor diagnostic criterion [2]. Noncontrast chest CT is generally sufficient in the detection of small septic emboli within the lung parenchyma. Contrast-enhanced CT, (^18^F)FDG PET/CT, and WBC SPECT/CT well may detect pulmonary consolidations and discriminate septic infarcts and abscesses from neoplastic lesions. The use of ventilation-perfusion scintigraphy has been replaced by other techniques, particularly (^18^F)FDG PET/CT and WBC SPECT/CT, which have the clear advantage to combine the detection of distant embolism, the assessment of valve-involvement, and identification of POE [31].

### 5.6. Role of CMR in Diagnosing IE and Local Complications

As for the left valves, the role of CMR in the diagnosis of IE and local complications is poorly codified and limited to selected cases, where the other imaging techniques are not sufficient or contraindicated (e.g., renal failure or pediatric population in substitution of CCTA).

Nevertheless, CMR may offer a superior evaluation of right transvalvular flows compared to TEE by using phase contrast imaging, which is particularly useful to quantify pulmonary valve regurgitation caused by perforations or destruction of the cusps or the abnormal flow of paravalvular leakage [41].

### 5.7. Diagnostic Workflow Summary

• Similarly to left-sided native IE, the workflow of suspected right-sided IE is based on TTE, TEE, and blood cultures to stratify patients according with Duke criteria. TTE has a central role in exploring tricuspid valve, while TOE is generally required in the case of pulmonary valve involvement.
• Second-line imaging (CT, PET/CT, WBC SPECT/CT) is indicated to search for perivalvular and pulmonary complications, to detect distal embolisms, and to identify POE.

## 6. Early and Late Prosthetic Valve Infective Endocarditis (PVE)

### 6.1. Main Clinical Characteristics

IE is one of the most challenging complications after heart valve surgery, often requiring complex diagnostic work-up and affecting up to 6% of patients with prosthetic valves [42]. PVE or post-repair endocarditis accounts for the 30% of cases in Euro-Endo registry [21], with 18.1% of patients having a history of previous endocarditis. Traditionally, PVE is classified as early PVE if infection occurs within 12 months from surgery and late PVE if it occurs thereafter [2]. In early PVE, patients are generally infected perioperatively and, more frequently, can present complications involving the sewing ring (abscess, dehiscence, pseudoaneurysms, and fistulae), with Staphylococci, fungi, and Gram-negative bacilli as the main responsible germs [43]. Late PVE generally occurs with valve regurgitation if a bioprosthesis is involved, while mechanical valves may present with large vegetations causing valve stenosis [44]. The germs responsible for late PVE are substantially the same involved in native valve IE.

PVE presents the highest in-hospital mortality (20–40%) among IE [45]. Older age, diabetes mellitus, healthcare-associated infections, staphylococcal or fungal infection, early PVE, HF, stroke, and intracardiac abscess are the main risk factors associated with poor prognosis [46]. The high mortality and the complex diagnostic work-up require an early treatment strategy, eventually including surgery, to control the disease burden. Surgery is mandatory in case of HF, severe prosthetic dysfunction, abscess, or persistent fever [2]. A conservative strategy with close follow up is indicated for the low-risk cases.

### 6.2. When to Ask for TTE and When to Ask for TOE

**Figure 5:**
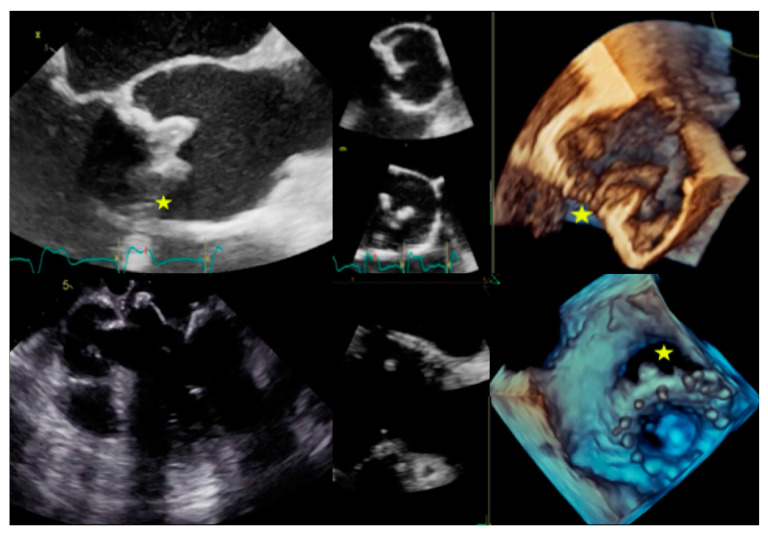
Complications of prosthetic valve endocarditis (PVE). Top: Biological prosthesis dehiscence in aortic position with large perivalvular leak (left: TOE long-axis view, right: 3D TOE view). Bottom: Biological prosthesis dehiscence in mitral position with large perivalvular leak (left: TOE long-axis view showing the direct communication between atrium and ventricle, right: 3D TOE view). Stars indicate the place of maximal prosthesis dehiscence.

As for native IE, the suspicion of PVE requires TTE and TOE evaluation. Unfortunately, false negative examinations are more frequent and limit the accuracy of the echocardiography [4]. The TOE is generally preferred as a first-line evaluation and it is also indicated in case of negative TTE, in both early and late PVE, and for the identification of periprosthetic abscess and leak (both major Duke criteria, Figure 5).

The Euro-Endo Registry showed that TOE is generally performed in the 66% of subjects, showing a significant percentage of abscesses (19%), pseudoaneurysm (6%), and prosthetic dehiscence (11%) [21]. The limited sensitivity of Duke criteria in PVE supports the indication to repeat TOE when the clinical suspicion is high, according with the clinical evolution [47].

### 6.3. Role of CCTA in Diagnosing PVE and Local Complication

CCTA imaging helps diagnose PVE when results of TTE or TOE are indeterminate or to assess paravalvular complications if the PVE diagnosis has already been established [2]. In PVE, the infection usually spreads from the sewing ring or adjacent thrombi and may result in complications such as paravalvular leakage, abscess, pseudoaneurysm, dehiscence, and extension to adjacent structures [44].

Despite the beam-hardening artifacts, due to the metal component of the prosthesis, which affect image quality and may hinder the visualization of small vegetations (<4 mm), vegetations larger than 10 mm are usually detected with high accuracy by CCTA. They typically appear as round, mobile, hypodense masses on the valve leaflet or sewing ring, typically on the ventricular side of aortic or mitral leaflets [48].

CCTA is more sensitive than TOE in detecting paravalvular and extracardiac infection involvement [48] and should be acquired with retrospective gating with subsequent reconstruction of every 10% RR interval of the entire cardiac cycle to enable cinetic CT visualization.

Paravalvular abscesses are well recognized by CCTA as a thickened, hypoattenuating area or irregular, inhomogeneous mass surrounding the PV or the aortic root, inconstantly associated to rim delayed enhancement and gas bubbles, reflecting infected cavities adjacent to the PV.

Extent of inflammation to surrounding tissues is depicted by adjacent fat stranding or, exceptionally, by myocardial thickening and enhancement, or mediastinal gas and fluid collections when the infection spreads outside the pericardial sac.

**Figure 6:**
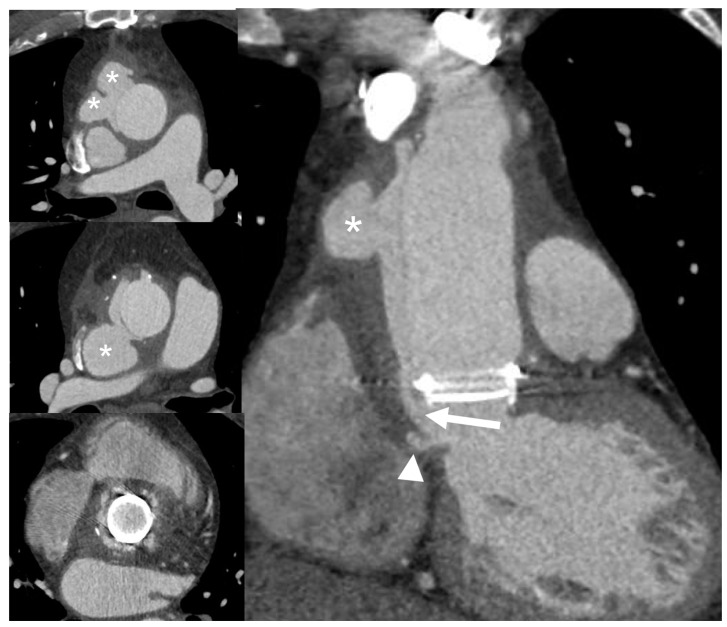
Infective late complication in a patient with Bentall prosthesis: Dehiscence and periprosthetic pseudoaneurysm. CCTA multiplanar reconstructions show a large dehiscence of the surgical suture at the proximal anastomosis of the prosthesis with large communication (arrow) of the left ventricular cavity with a large false lumen (asterisks) recanalized from perivalvular communication (arrow); a little periannular pseudoaneurysm (arrowhead) is seen adjacent to the ventriculo-aortic junction.

Pseudoaneurysms are a typical late complication after valve surgery during aortic root graft replacement or Bentall procedure and they can occur when a perivalvular cavitating abscess drains into the adjacent cardiac chamber, with the formation of wall outpouching containing circulating blood (Figure 6).

PVE may be complicated by perforation of leaflets (exclusively in biological prostheses) or dehiscence, defined detachment of the PV from its annulus due to rupture of the suture line between the sewing ring and annular tissue. CCTA imaging, including cine-CT reconstruction, may visualize the rocking motion from a severely detached prosthesis. PVE-related dehiscence may also cause paravalvular leakage with abnormal communication between two different chambers through the hole at the undocking point.

Another potential complication, which can be demonstrated by CCTA, is the formation of fistula connecting two neighboring cavities (such as a Valsalva sinus with right ventricle or left atrium or between the left ventricle and right atrium); in this setting CCTA offers a detailed morphological visualization of the abnormal connections.

Finally, CCTA improves the precision of radionuclide imaging study by providing an anatomical map co-registered with functional information provided by metabolic imaging [49].

### 6.4. When to Ask for Nuclear Imaging

**Figure 7:**
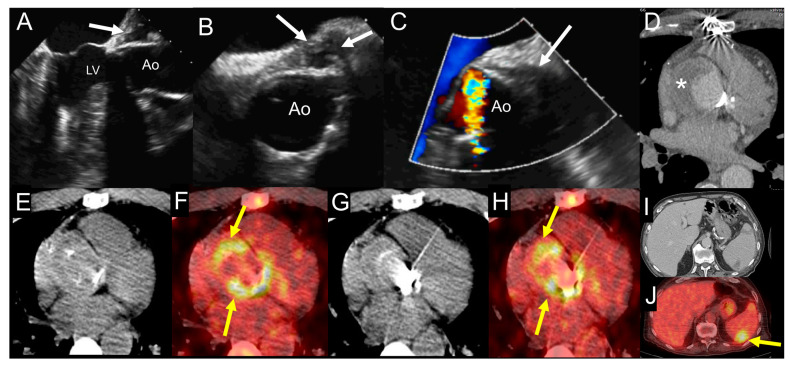
Example of the use of multimodality imaging. A patient with history of aortic valve replacement with mechanical prosthesis and ascending aorta graft presented four years later with acute right lower limb ischemia due to occlusion of proximal fibular, anterior tibial, and posterior tibial arteries treated with revascularization attempts and finally leg amputation. During hospitalization, the patient had fever with increased erythrocyte sedimentation rate and C-reactive protein. TTE (**A**) and at TOE (**B**,**C**) show the presence of hyperechogenic periprosthetic area (white arrows), most likely consistent with abscess. Blood culture was negative. The fluoro-18-fluorodeoxyglucose positron emission tomography/computed tomography ((^18^F)FDG PET/CT) exam including CCTA (**D**-**J**) was performed showing an organized fluid perigraft collection surrounded by thick walls (asterisk) that enhance after iodinated contrast injection on CCTA images (**D**), which is associated to intense uptake of (^18^F)FDG around the aortic valve prosthesis, as shown by the yellow arrows ((**E**,**G**) show noncontrast CT transaxial images while (**F**,**H**) show the fused PET/CT images). Myocardial suppression of (^18^F)FDG uptake is achieved by high-fat, low-carb diet. In addition, the whole-body images showed an area of spleen uptake, consistent with septic embolism ((**I**,**J**), noncontrast CT and fused PET/CT transaxial images, respectively), as indicated by the yellow arrow. Ao: ascending aorta; LV: left ventricle.

In the case of prosthetic valves, “abnormal activity around the site of implantation” detected by (^18^F)FDG PET/CT is a major criteria for IE according to ESC 2015 guidelines [4]. Therefore, all patients with possible or even rejected IE by Duke criteria, but with high clinical suspicion, should be assessed by (^18^F)FDG PET/CT and/or WBC imaging or CCTA to confirm/rule out IE (Figure 7) [31]. Conversely, patients with rule-out IE according to Duke criteria and with low clinical suspicion do not require further examinations.

Several recent meta-analyses indicated that the overall pooled sensitivity of (^18^F)FDG PET/CT in PVE is about 73–81% [50] with an overall accuracy with an area under curve (AUC) of 0.897 when including only studies reporting adequate cardiac preparation. Even in the case of negative PET results (that includes also a whole-body evaluation for embolism detection), a thorough interpretation of the echocardiography and CCTA scan is essential. Indeed, in the absence of infection, PV generally shows mild, diffuse, and homogeneous uptake that usually remains stable for at least one year after surgery, most likely resulting from persistent host reaction against the biomaterial coating the sewing ring of PV and chronic tension or friction exerted on these anchor points [51,52,53]. Such (^18^F)FDG uptake seems to be slightly greater in mechanical versus biological prostheses.

Very recent data proved the prognostic value of (^18^F)FDG PET/CT in PVE. In a large retrospective study on 173 patients with left-sided IE and examined after seven days from the first antibiotic administration, a moderate to intense valvular (^18^F)FDG uptake was predictive of major adverse cardiac events defined as in-hospital death, one-year death, recurrence of IE, acute cardiac insufficiency, symptomatic embolism under antibiotics, and nonscheduled rehospitalization for cardiovascular indication [54]. These results reinforce the utility of (^18^F)FDG PET/CT in PVE and justify its use in this population, not only for diagnostic purposes, but also for prognostic assessment. Therefore, (^18^F)FDG PET/CT should be used in clinical practice for optimal patient management and therapy decision making, particularly in PVE.

### 6.5. How to Search for Embolisms

Notable advantages of PET/CT and WBC SPECT/CT are their ability to perform the extra-cardiac workup within a single imaging procedure and to reveal the concomitant presence of extra-cardiac infection sites as the consequence of both septic embolism as well as primary infective processes (with the exception of the brain location, since brain uptake is always high due to its specific metabolism) [28,55,56]. Detection of metastatic infection by (^18^F)FDG PET/CT leads to change of treatment in up to 35% of patients [57]. PET/CT has demonstrated to be able to reveal the source of infection, including cases where the sustaining POE was a neoplasia (colonic cancer) [6]. As for IE of native valves, with distant septic embolization a minor criterion for PVE diagnosis, their search depends on the left or right side of the prosthetic valve and includes also cerebral MRI and whole-body, contrast-enhanced CT, as previously described for native valves.

### 6.6. Role of CMR in Diagnosing PVE and Local Complications

CMR is affected by metal artifacts especially from mechanical prostheses and offers similar information to the CCTA (including detection of paravalvular leakage, abscess, pseudoaneurysm, and dehiscence) but with lower spatial resolution and lower anatomical definition. It is typically used when CCTA is contraindicated or to assess complex hemodynamic, such as to ascertain or evaluate intracardiac fistula where CMR is able also to quantify the shunt.

### 6.7. Diagnostic Workflow Summary

• As for native valve IE, in case of suspected PVE the initial assessment is based on Duke criteria, with the specific indication to perform both TTE and TOE for the higher accuracy of transesophageal approach.
• If IE is rejected and the suspicious is low, no more investigations are needed.
• If the diagnosis is definite, the patient should be investigated for silent embolism or metastatic infections using CT or PET/CT scan and with CCTA to detect periprosthetic extension. MRI to detect cerebral involvement (embolism or hemorrhage) is also indicated according with the clinical status.
• In case of possible IE or rejected IE but high suspicion, there is indication for repeating new TTE/TEE and blood cultures. CCTA or PET/CT are recommended to detect periprosthetic extension. A whole-body, contrast-enhanced CT or PET/CT or WBC SPECT/CT is indicated to detect silent embolism or metastatic infections. All these methods contribute to reclassify the patient according with ESC 2015-modified diagnostic criteria [2].

## 7. CIED-Related Infective Endocarditis

### 7.1. Main Clinical Characteristics

CIED-IE represents about 10% of total IE, with an in-hospital mortality of 15.3%, according with Euro-Endo Registry [21]. A wider use of CIEDs in the elderly contributes to the increased rate of CIED-IE, with an incidence of 1.9 per 1000 device/year reported in a population-based study [58]. Clinically, CIED-IE can be divided into infections limited to the pocket of the skin and infections extended to the electrode leads, cardiac valve leaflets, or endocardial surface. This difference is challenging to define and can require the use of advanced imaging, such as WBC scintigraphy or (^18^F)FDG PET/CT [59].

The infection can primarily involve the pocket, after direct manipulation (i.e., change of generator) and spread to the leads producing multiple vegetations or it can directly originate on the leads during bacteremia, secondary to a distant site of infection. In addition to the typical risk factors for IE (renal failure, corticosteroid use, congestive HF, and diabetes mellitus), other factors related to the surgical procedure may play a role in CIED-IE (i.e., type of intervention, device revision, use of temporary pacing, use of antimicrobial prophylaxis, and use of anticoagulation) [60]. *Staphilococci* accounts for 60–80% of the cases, with a significant proportion of *S. aureus* (about 50%), according with the Euro-Endo registry [21].

From a clinical perspective, it is important to differentiate superficial incisional infection, which does not require CIED system extraction [61,62], from infection limited to the pocket, extending to the leads potentially associated with systemic infections and/or IE.

### 7.2. When to Ask for TTE and When to Ask for TOE

The clinical manifestation of CIED-IE can be variable and misleading, with acute manifestations or chronic evolution, especially in the elderly. As for other specific conditions of IE, the TTE and TOE play a first-line role for searching vegetation in the intracardiac and intravascular portions of the leads. The diagnostic usefulness of echocardiography is limited to the infections involving the explorable portions of the leads (i.e., the intracardiac and superior vena cava initial segments), having a negligible role in pocket-related infections. TOE has higher sensitivity than TTE in identifying the tricuspid involvement, the presence of vegetation on the leads, and the involvement of left-sided valves [63]. TTE allows for a better assessment of pulmonary pressures, pericardial effusion, and left ventricle function. Both approaches are, therefore, recommended in case of suspected CIED-IE. The echocardiography should be used to identify the vegetation and its localization, but a negative examination does not rule out the presence of infection. The Euro-Endo Registry showed that a high proportion (67%) of patients with CIED-IE received TOE, but a significant percentage of subjects (26% and 37%, respectively) also required PET/CT or chest CT [21]. This observation confirms the need for multidisciplinary work-up in CIED-IE.

### 7.3. Role of CCTA in Diagnosing CIED-IE and Local Complications

In CIED-IE, CCTA has poorer sensitivity in detection of vegetations on pacemaker leads compared to TTE or TOE, due to blooming and beam-hardening artifacts and should be limited to situations when radionuclide imaging is not available [31].

Electrocardiographic (ECG)-gating embedded in CCTA refines the anatomical map co-registered during radionuclide imaging study, improving the diagnostic accuracy of hybrid exams [64].

Moreover, CCTA can be followed by a nongated, contrast-enhanced CT scan, which plays a role in assessing infection of the pacemaker pocket by depicting local inflammatory tissue changes or abscess collection around the device, which should be distinguished from non-infected hematomas, superficial cellulitis, or infection that commonly occurs at the surgical site in the postoperative phase [65].

Contrast-enhanced CT is also required in the detection of distant septic emboli and predominantly pulmonary and vascular complications such as mycotic aneurysms. This represents additional criteria for CIED-IE diagnosis with direct impact on patient management and treatment strategy.

### 7.4. When to Ask for Nuclear Imaging

**Figure 8:**
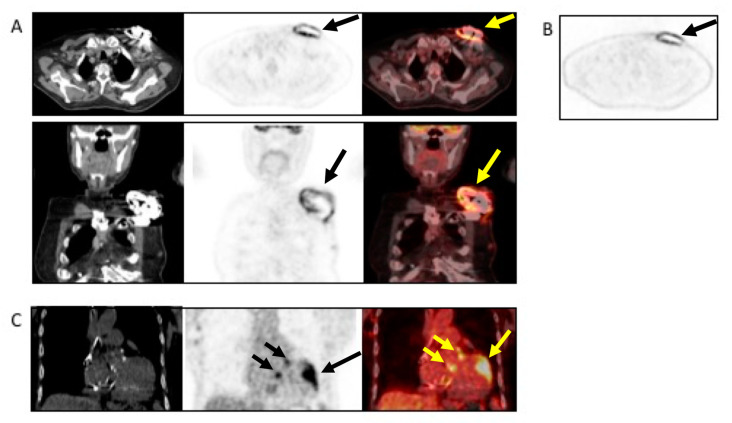
Example of the use of (^18^F)FDG PET/CT in a patient with non-Hodgkin’s lymphoma and sudden onset of fever and a positive blood culture for *Streptococcus dysgalactiae.* The patient underwent TTE and TEE, which were negative. Antimicrobial treatment was started. Due to the lack of clinical response, the patient underwent (^18^F)FDG PET/CT, which revealed infection, as indicated by the black and yellow arrows, involving the pocket of the device ((**A**), from left to right, CT, PET, and superimposed PET/CT transaxial (upper panel) and coronal (lower panel and (**B**) non-attenuated corrected transaxial images) as well as the intracardiac portion of the lead extending to the tricuspid valve ((**C**), from left to right, noncontrast CT, PET, and fused PET/CT coronal images). Based to the PET/CT findings, the device was extracted and replaced.

(^18^F)FDG PET/CT provides added diagnostic value to the Duke criteria, particularly in the subset of possible CIED-IE [66,67,68,69,70,71] and it has the capability to explore the whole device. Therefore, (^18^F)FDG PET/CT and WBC SPECT/CT might be used in all the cases when CIED involvement is suspected [8]. In CIED-IE the presence of (^18^F)FDG uptake should be described as pertinent to generator pocket (superficial or deep) and/or to the leads (intravascular or intracardiac portion of the leads). In addition, signs of cardiac (valvular or pericardial) involvement as well as systemic signs of infections (septic embolism, in particular in the lung parenchyma and POE) should be carefully assessed and reported. The (^18^F)FDG PET/CT is useful in patients with evidence of pocket infection and negative microbiologic and echocardiographic examination and in patients with positive blood cultures but negative echocardiographic examination. All the related studies have shown an almost 100% accuracy for infection of the generator pocket and for the extracardiac portion of the lead (sensitivity, specificity, and accuracy for the diagnoses of pocket infection were 93%, 98%, and 98%, respectively) [31,50,72]. The presence of (^18^F)FDG uptake along pacing leads, in particular, in the same location as mobile elements on echocardiography and in association with septic pulmonary emboli appearing as multiple focal (^18^F)FDG spots, is highly suggestive of pacing lead infection [67] (Figure 8). Of notice, in the Euro-Endo registry extracardiac uptake was found in 43.8% of patients with CIED-IE [14].

In the case of lead-related IE, (^18^F)FDG PET/CT is very specific when tracer uptake is visualized. However, its sensitivity is low, and a negative result does not completely exclude the presence of small vegetations with low metabolic activity [68]. Every positive blood culture should be carefully evaluated, with prompt, active exclusion of CIED-IE with other diagnostic techniques [8].

### 7.5. Diagnostic Workflow Summary

• In case of suspected CIED-IE, the physician should address the Duke criteria, being aware of the significantly limited diagnostic accuracy. However, blood cultures, TTE, and eventually TOE should be considered also in case of suspected infection limited to the pocket.
• In case of possible CIED-IE or rejected CIED-IE but persistent high clinical suspicion there is indication to repeat TTE/TOE and blood cultures.
• Chest CT has a specific role in searching for pulmonary embolism, infarct or abscess. The (^18^F)FDG PET/CT and WBC SPECT/CT have a central role in detecting pocket infection, lead infection, and pulmonary embolism.

## 8. Current Challenges and Future Perspectives

The diagnosis of IE and CIED-IE still remains sometimes a challenge for the clinicians for both diagnostic and therapeutic points of view. As previously shown, each imaging modality has its own pros and cons (Table 1) and, therefore, the appeal to an integrated and multimodal approach in the diagnostic workup of IE and CIED-IE is mandatory. It has already demonstrated to be effective for the early identification of the infection. But, at the moment, the role of imaging as tool to follow-up after antibiotic therapy or in decision making between a surgical rather than a medical approach is still debated and the lack of a reference standard represents one of the most critical aspects that should be faced in the near future.

Novel trends in radiopharmaceuticals’ developments as well as significant progresses in technology and new insights on the various mechanisms that play a role in cardiovascular infections will likely provide in the future new diagnostic and therapeutic targets for further developments in the field.

As for the radiopharmaceutical perspective, while radiolabeled granulocytes are a common clinical practice with SPECT applications, tracking WBC in vivo with PET using the positron emitting is still in the research phase [73,74,75]. A very interesting innovative strategy, based on the development of selective metabolic probes that are substrate for specific strains, has recently renewed the interest in pathogen-specific imaging agents. In fact, while traditional approaches have been based on radiolabeling existing antibiotics (i.e., ciprofloxacin) or antimicrobial peptides (i.e., ubiquicidin), researchers have recently tested almost 1000 radiolabeled small molecules as substrates for essential metabolic pathways in bacteria, demonstrating (^18^F)fluorodeoxysorbitol [76,77] holds tremendous potential for identifying and monitoring known or suspected infection caused by *Enterobacteriaceae*. On the other hand, in addition to technological developments, new equipment such as PET/MRI and total-body PET/CT will provide new opportunities to extend clinical diagnosis in specific scenarios (i.e., myocarditis) as well as to implement the use of quantitative imaging analysis. All together, this synergy arising from the combination of clinical and technological aspects represents the next challenge to unravel the full potential of multimodality imaging into daily clinical practice of patients with cardiovascular infections.

## 9. Conclusions

A modern and updated management of IE and CIED-IE requires a correct and synergistic integration of diagnostic tools and therapeutic strategies. Multimodality imaging is a crucial part of diagnostic work-up where different techniques provide additional and unique information. Clinicians and imaging specialists should be aware of strengths and limitations of every approach in order to correctly interact with other specialists and, therefore, to optimize the management of patients and to improve the outcome.

## Figures and Tables

**Table 1:** Multimodality imaging in the assessment of patient with IE, PVE, or CIED infection.

	Echocardiography		CCTA		PET/CT		WBC SPECT/CT		CMR	
	Pro	Cons	Pro	Cons	Pro	Cons	Pro	Cons	Pro	Cons
**General Comments**	The first-line diagnostic tool. Diagnostic significance: providing information major Duke/ESC criteria. Prognostic significance: complication and prediction of the risk of embolism Able to assess treatment response. Widely available and unexpensive. TTE can be easily repeated.	Diagnostic accuracy of TTE/TOE is operator-related. TOE requires patient sedation, not always feasible. Limiting factors: poor acoustic window (COPD, thorax conformation), artifacts due to calcium/metals.	Diagnostic significance: major ESC criteria. Possibility to study coronary arteries at the same time. Prognostic assessment: embolisms detection with whole body contrast enhanced CT scan. Wide availability.	Radiation exposure. Risk of contrast-induced nephropathy.	Combination of metabolic evaluation and anatomic assessment. Diagnostic significance: major ESC criteria. Prognostic assessment: Simultaneous detection of embolism, metastatic lesions, portal of entry. Good availability. Easy to perform. Possibility to combine with CCTA evaluation of coronary tree at the same time.	Radiation Exposure. Patient preparation for myocardial suppression. If iodinate contrast is not administrated limited value for brain assessment. Prolonged antimicrobial treatment reduce intensity of [^18^F]FDG uptake. Pattern of uptake is important.	Combination of metabolic and anatomic assessment. High specificity for infection. Diagnostic significance: major ESC criteria. Prognostic assessment: simultaneous detection of embolism, metastatic lesions, portal of entry.	Radiation Exposure. Need of blood manipulation. Limited sensitivity for small lesions. Relative complex procedure. Low availability. Long acquisition time.	Absence of ionizing radiation. It can offer diagnostic images even without using contrast medium (can replace CCTA in patients with renal failure). It offers morphological and functional information (i.e., valve dysfunction, shunt quantification).	Sensitive to breath artifacts (good patient compliance required). Intermediate availability. Long acquisition time.
**Left-sided IE**	Good visualization of mitral and aortic valve. Valvular dysfuction assessment. Identification of complication (i.e., valvular regurgitation).	Difficult differential diagnosis in presence of marantic vegetations or high calcification.	Detection of vegetations and valve perforation. Assessment of perivalvular extent of disease (abscesses, pseudoaneursysm, fistula).	Inferior to TTE/TOE in detecting small vegetations (<2 mm).	Prognostic assessment: simultaneous detection of embolism, metastatic lesions and portal of entry.	Limited sensitivity for small vegetations.	Evaluation of distant emboli and portal of entry.	Limited role because of low sensitivity for small vegetations.	Capability to assess vegetations (inferior to TTE/TOE). Capability to assess local complications. Independent by acoustic window. May detect concomitant myocardial inflammation.	Not included in current guidelines for IE diagnosis.
**Right-sided IE**	TTE generally provides good visualization of tricuspid valve. TOE is useful in the assessment of IE related to CHD.	Pulmonary valve is difficult to assess.								
**PVE**	Routinely used for follow up; it allows sequential assessment of prosthesis function. TOE is often required to correctly assess the prosthesis.	Limited by prosthetic material artifacts (i.e., acoustic shadow). Early complication (i.e., abscess) can be difficult to identify.	Identification of complications (paravalvular leakage, abscesses, pseudoaneurysm, dehiscence, and extension to adjacent structures). Capability to visualize large vegetations (>10 mm).	Low image quality for beam hardening artifacts. Limited in assessing small vegetations (<4 mm).	High diagnostic accuracy. Good assessment of perivalvular/periprosthetic complications. Reduction of rate of misdiagnosed PVE. Role in prediction of MACEs. Prognostic significance.	Host reaction may reduce specificity (risk of false-positive studies until 3 months after surgery).	High specificity for infection. Reduction of rate of misdiagnosed PVE. Differential diagnosis between septic and sterile vegetations.	Limited sensitivity for small lesions.		Image quality severely hampered by susceptibility artifacts (especially from mechanical prostheses).
**CIED-IE**	Useful to assess intracardiac lead segments. TTE can be integrated by ultrasound evaluation of device pocket, to assessing inflamation or fluid collection.	Limited role in the assessment of unexplorable lead segments. Differential diagnosis of vegetation vs. lead fibrosis/thrombi can be challenging.	Possibility to combine the CT assessment of generator pocket.	Blooming and beam hardening artefacts. Poor sensitivity in detecting vegetations on leads.	Very high sensitivity and specificity for generator/pocket and extracardiac or extravascular lead infection.	Low sensitivity for small vegetations along the leads.	Good sensitivity and specificity for generator/pocket and extracardiac or extravascular lead infection.	Limited diagnostic sensitivity for intracardiac and intravascular lead infection.		Image quality severely hampered by susceptibility artifacts from lead and device. Limited to patients with MRI conditional devices and with numerous precautions.

IE: Infective Endocarditis; PVE: Prosthetic Valve Endocarditis; CIED-IE: Cardiac Implantable Electronic Device-related infective endocarditis; CCTA: Cardiac Computed Tomography; PET/TC: Fluoro-18-fluorodeoxyglucose positron emission tomography/computed tomography; WBC SPECT/CT: radiolabelled white blood cells scintigraphy with single-photon emission computed tomography/computed tomography; CMR: Cardiac Magnetic Resonance; TTE: trans-thoracic echocardiography; TOE: trans-oesophageal echocardiography; CT: computed tomography; IE: infective endocarditis; COPD: Chronic obstructive pulmonary disease; CHD: congenital heart disease; MACEs: major adverse cardiac events; CIED-IE: cardiac implantable electronic device-related infective endocarditis; MRI: magnetic resonance imaging.
